# Animal Species and Identity Testing: Developments, Challenges, and Applications to Non-Human Forensics

**DOI:** 10.3390/genes16121503

**Published:** 2025-12-16

**Authors:** Bruce Budowle, Antti Sajantila, Daniel Vanek

**Affiliations:** 1 Department of Forensic Medicine, University of Helsinki, 00014 Helsinki, Finland; 2 Forensic Medicine Unit, Finnish Institute for Health and Welfare, 00271 Helsinki, Finland; 3 Institute for Environmental Sciences, Charles University, Benatska 2, 12800 Prague, Czech Republic; 4 Forensic DNA Service, Budinova 2, 18081 Prague, Czech Republic; 5 Bulovka University Hospital, Budinova 2, 18000 Prague, Czech Republic; 6 Department of Forensic Medicine, Second Faculty of Medicine, Charles University, 11000 Stare Mesto, Czech Republic

**Keywords:** CITES, species identification, individual identification, DNA barcoding, databases

## Abstract

Biological samples of non-human origin, commonly encountered in wildlife crime investigations, present distinct challenges regarding forensic DNA analysis efforts. Although the types of samples encountered in human identity testing can vary to some degree, analyzing DNA from one species is facilitated by unified processes, common genetic marker systems, and national DNA databases. In contrast, non-human animal species identification is confounded by a diverse range of target species and a variety of sampling materials, such as feathers, processed animal parts in traditional medicine, and taxidermy specimens, which often contain degraded DNA in low quantities, are contaminated with chemical inhibitors, and may be comingled with other species. These complexities require specialized analytical approaches. Compounding these issues is a lack of validated non-human species forensic sampling and typing kits, and the risk of human DNA contamination during evidence collection. Markers residing on the mitochondrial genome (mtDNA) are routinely sought because of the large datasets available for comparison and their greater sensitivity of detection. However, the barcoding results can be complicated at times for achieving species-level resolution, the presence of nuclear inserts of mitochondrial DNA (NUMTs), and the limitation of mtDNA analysis alone to detect hybrids. Species-specific genetic markers for identification have been developed for a few high-profile species; however, many CITES (Convention on International Trade in Endangered Species of Wild Fauna and Flora)-listed organisms lack specific, validated forensic analytical tools, creating a significant gap in investigative enforcement capabilities. This deficiency stems in part from the low commercial nature of wildlife forensics efforts, a government research-driven field, the difficulty of obtaining sufficient reference samples from wild populations, limited training and education infrastructure, and inadequate funding support.

## 1. Introduction

The challenges inherent in non-human identity testing create a critical resource gap in the development of specific, validated tools needed for many Convention on International Trade in Endangered Species of Wild Fauna and Flora (CITES)-listed organisms. These forensic limitations impact investigations by complicating species attribution, the assessment of genetic diversity, individual identification for population management and forensic case-specific questions, and the confirmation or exclusion of hybrids. These challenges are compounded by extensive inbreeding common in small, endangered populations. Addressing these gaps is essential for strengthening enforcement capabilities and supporting effective species protection initiatives.

Forensic investigations involving non-human biological material, particularly in wildlife poaching and trafficking, demand a specialized analytical framework [1,2]. Twenty years ago, Budowle et al. [3] proposed recommendations for animal forensic DNA and identity testing covering analytical practices, data evaluation, nomenclature, allele designation, statistics, validation, proficiency testing, lineage markers, casework files, and reporting to address the missing guidelines for this sub-area of identification genetics. The International Society of Forensic Genetics (ISFG) built on this need and published a comprehensive suite of recommendations in 2011, further formalizing best practices for non-human (animal) DNA in forensic genetic investigations [4]. Forensic genetics laboratories that analyze non-human species rarely focus on a single species, and thus the analytical demands may seem insurmountable, including diverse sample types, nondestructive or nominal damage to precious artifacts, insufficient reference datasets, lack of dedicated marker sets, understanding species-specific genetics, nominal laboratory resources and expertise, to name a few.

Several papers have outlined the significant challenges faced in wildlife forensic science [5,6,7]. The samples range from animal parts in traditional medicine and veterinary medicine [8] to taxidermy and environmental specimens to animal-derived food products, and they often contain degraded or low-quantity DNA that can be contaminated with chemical inhibitors and may be comingled with other species. The application of methods and tools designed for human DNA forensics may be insufficient for the diverse and complex nature of wildlife evidence [9,10]. But technologies and bioinformatics continue to improve, and substantial inroads have been made to address these analytical challenges.

There are species beyond animals that can be considered as well, such as plants and particularly microorganisms, which are increasingly relevant in investigations of biocrime, bioterrorism, estimating postmortem interval, provenance, and human identity cases. However, herein, the focus will be on an overview of the challenges of identifying animal species. While previous overview papers have addressed the challenges of forensic investigations on wildlife-related crimes (see Smart et al. and Johnson et al. [11,12]), it is also necessary to consider the above-mentioned challenges from the perspective of the objective of analysis and the types of samples involved.

## 2. Challenges in Wildlife Genetic Identity Testing

**Figure 1:**
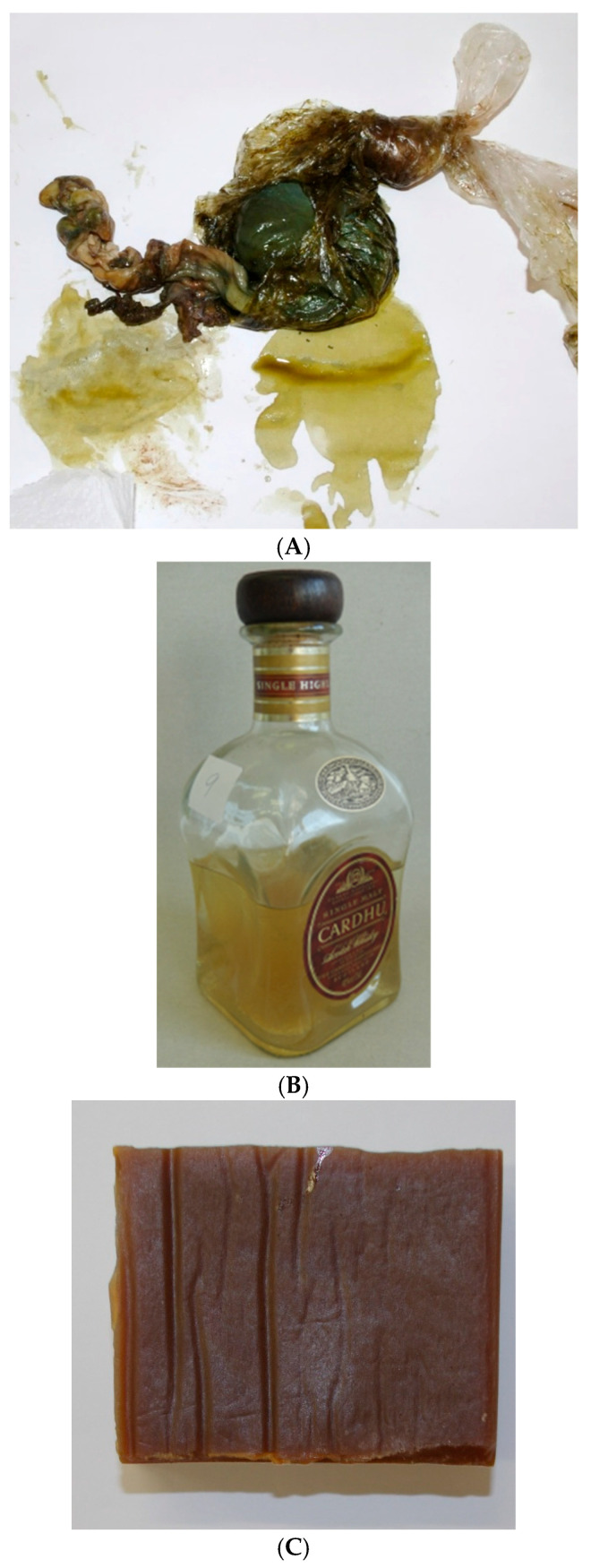
(**A**) Bile duct from *Pantera tigris.* (**B**) “Tiger bone wine” where the presence of *P. tigris* biological material was confirmed by DNA analysis. (**C**) An example of a “Bouillon cube” made from bones.

Highly endangered species are of particular interest from environmental protection, species survival, economic, health, and identifying trafficking routes perspectives. Additionally, some traditional medicines are composed of materials derived from highly endangered species [13,14,15,16]. Taxidermy, the art of preserving, stuffing, and mounting the skins of animals, especially vertebrates, for display or study, may be performed on restricted animals. Dermoplasty (i.e., stuffed, tanned skin (or head) of an exotic animal placed on display) historically represented power, strength, and bravery [17], and thus was practiced for demonstrating status. Artifacts can be very challenging to identify due to many uncertainties regarding their composition and processing methods. Examples of such artifacts are shown in Figure 1A–C.

In the case of endangered species, trade is highly regulated by CITES, and thus, supply demands are sometimes met by so-called “fake/faux taxidermy.” Professional taxidermists often use “spare parts” to increase an artifact’s value by filling in missing teeth, bones, or skin. This branch of taxidermy uses substitutive materials, like paper or textile, or combines the parts of different animals, creating in essence “chimeras” [18,19]. The well-known “Magdeburg unicorn”, assembled from the body parts of a narwhal (*Monodon monoceros*), wooly rhinoceros (*Coelodonta antiquitatis*), and wooly mammoth (*Mammuthus primigenius*) [20], is an example of fossil reconstruction [21].

Animal DNA analysis can also serve the broader society in cases of adulterated food products or substituted materials. Some examples are meat species identification [22,23] and Halloumi cheese milk authentication [24].

While samples and scenarios are diverse, there are common, routine questions to consider in typical wildlife DNA identification in which biological material is recovered from an item (an artifact or evidence). Some of these questions are:

(a) What is the goal, i.e., resolution needed for the specific case?
(b) Are the samples provided sufficient for analysis?
(c) Are the data probative?
(d) Does the sample contain biological material from an endangered species?
(e) Is damage to the artifact during sampling a constraint?
(f) Is the artifact made from only one animal?
(g) Was the material chemically treated?
(h) What extraction procedure(s) will be appropriate?
(i) Which genetic marker(s) should be tested?
(j) How should the data be interpreted?
(k) Are there specific population genetics aspects that should be considered?
(l) Does the background create noise?

### 2.1. Sampling

**Figure 2:**
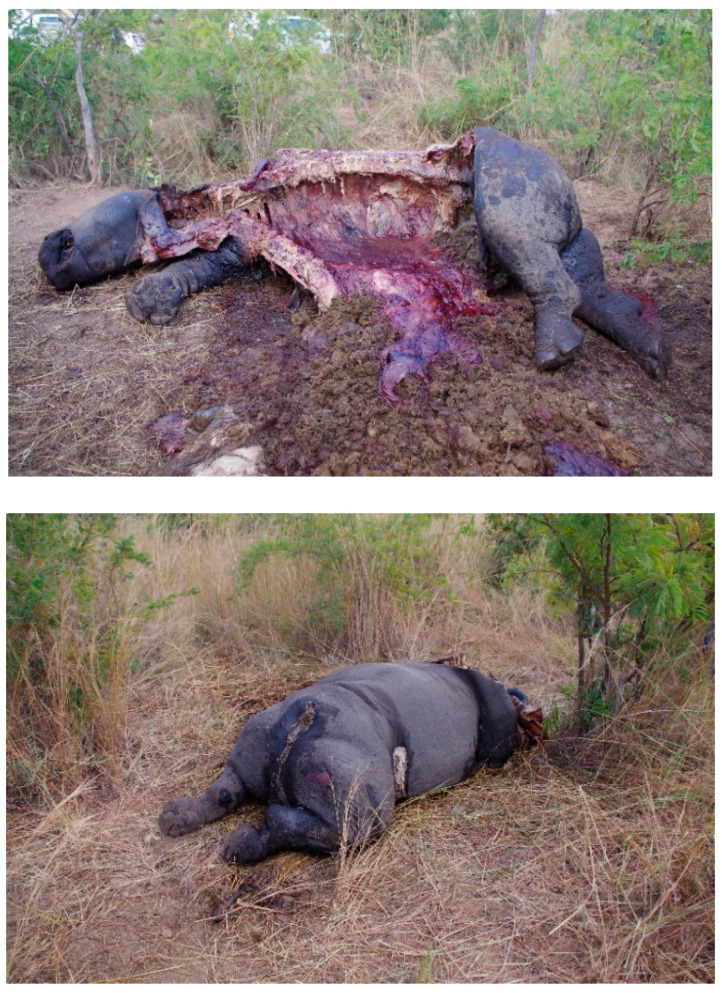
Poached female Rhinoceros (**upper**) with her baby (**lower**) hunted for the horn (photo taken at Kruger NP, South Africa, 2016).

The types of biological samples submitted for “identification” analysis can differ substantially from those routinely received for human identification (See Table 1). Herein, “identification” refers to the degree of attribution necessary for the case scenario. For human identification, the goal is individualization, although bioancestry and phenotype have been sought in some cases, but to a much lesser degree. For non-human identification, most often the goal, for example, with wildlife cases, is species-level resolution. The types of samples obtained in cases can include feathers, solid broth, “tiger bone wine”, processed skin and fur, and products of Traditional Chinese medicine (TCM) [25] (in the form of pills, powders, body parts, and tinctures [26]), tissue samples (Figure 2), processed meat, bushmeat [27,28], ornamental products, and residue (Figure 1A–C). They often contain low amounts of extractable DNA, can be substantially degraded due to putrefaction or the artifact preparation process (e.g., long boiling [29], use of chemicals in tanning [30]) often containing inhibitors, and can be mixtures of different species [31,32]. Some samples, such as hairs and feathers, inherently contain low levels of intact DNA and therefore should be collected in sufficient (herein, sufficient is not defined) quantity, when feasible.

**Figure 3:**
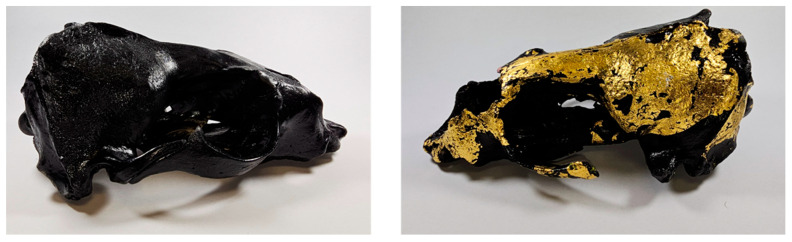
Painted skulls of the South Australian fur seal (*Arctocephalus forsteri*), seized at the Prague airport.

To minimize the likelihood of false-positive [33] or false-negative [34] results, sampling should be conducted from several parts of a composite artifact, when feasible or practical. Multiple sampling (as may be performed with human remains due to the heterogeneous distribution of DNA in a bone, as well as when dealing with secondary mass graves with commingled remains) is a good strategy in the case of, for example, tanned skin, as the chemicals used may not penetrate the skin equally, leaving some parts less treated and, therefore, less degraded and more extractable. Another common technique used by the taxidermist is the final coloration of the artifact (skin, bone, skull), which means additional sample processing may be necessary to remove such chemicals before DNA extraction (see Figure 3).

**Figure 4:**
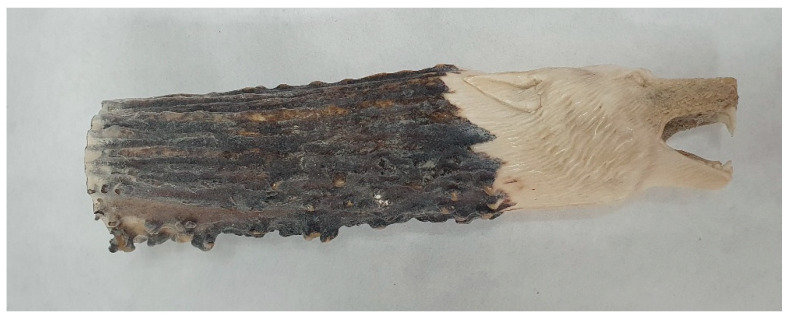
An artifact carved from the sambar (*Rusa unicolor*) antlers.

**Figure 5:**
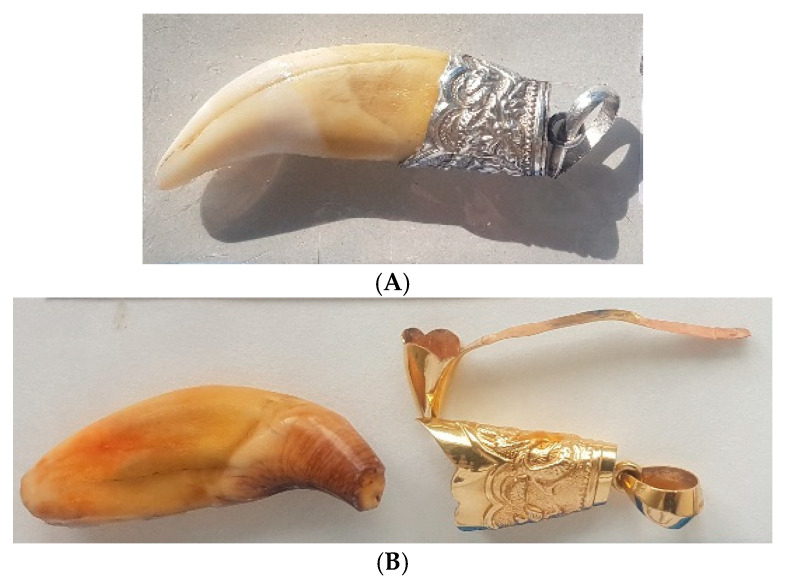
(**A**) Tooth of *P. tigris* mounted in silver. (**B**) Tooth of *Ursus thibetanus* mounted in gold.

Some artifacts submitted for species or individual identification also may have relatively high artistic, personal, or historical value (e.g., museum specimens). Thus, minimally destructive sampling should be employed [35,36]. Examples of such items are shown in Figure 4 and Figure 5.

### 2.2. Environmental DNA

Environmental DNA (eDNA), sampled from the air [37], water [38], or soil [39], may also be used in the forensic context [40,41,42] for covert sampling for operative purposes, animal tracking [43], or the detection of illegal wildlife trade [44]. The analysis of eDNA has been facilitated, particularly with advancements in high-sensitivity detection and rapid analysis. While not used for routine casework, this non-invasive method might help reconstruct events by showing where a person moved in a space or identify occupants in high-traffic areas where traditional touch DNA is too complex. Another eDNA use might be in linking individuals to a scene’s ecosystem. A finer detail than just soil type may be obtained, linking trace evidence to the unique ecology of a specific forest patch, field, or waterway. This type of tracking will require substantial validation studies and expansion of targets from microbes, fungi, and plants to create a detailed “biological fingerprint” of a location. There are also limitations to eDNA that must be considered, such as the high risk of contamination and confidence of attribution to a specific source (without individualizing methods).

### 2.3. Post-Sampling DNA Stability

The DNA (although degraded) is relatively stable in some samples by their nature, such as bones, teeth, feathers, and cured skins. For other samples, such as animal parts, meat, and fecal material, some stabilization and/or preservation measures are needed. Sampling kits containing reagents that stabilize DNA at ambient temperature and inactivate microorganisms are requisite, especially for fecal samples and food products. Preservation of the nucleic acids’ current integrity, especially when collected in the field where cold-chain storage is not available, can be maintained with stabilizing solutions or dry-down materials [45,46]. Such solutions should also inactivate infectious agents (viruses, bacteria, fungi, and parasites) [47,48] for health and safety (such as zoonotics [49,50]), as well as to prevent microbial degradation of forensic samples [51,52].

### 2.4. Human Contamination of Non-Human Samples

Wildlife genetic identity testing typically focuses on the DNA of the species of interest. But the person(s) preparing or handling the illicit items also may have left behind their own cellular material, which contains human DNA and can be an important analytical target for developing investigative leads regarding a perpetrator. Thus, special care must be taken not to contaminate the sample with human DNA during collection. This constraint is exacerbated when sample collection is performed by personnel not trained in forensic handling and sampling techniques, such as environmental inspectors or customs officers. Training and equipping investigators with PPE and proper handling protocols are tantamount.

The reagents and supplies used for human identification are typically manufactured to be [human] nuclear-DNA-free and are governed by ISO 18385 [53]. This norm does not address animal DNA as a possible contaminant in manufactured tubes, swabs, and sampling kits that are used in non-human forensic analyses [54,55]. Even the reagents used for PCR amplification can be a source of non-human DNA contamination, which is a leading cause of false positives [56,57,58]. One relatively efficient solution could be treating reagents with heat-labile dsDNase, but it does have several limitations [59]. Human DNA contamination need not confound analysis of a single-source animal sample subject because species-specific primers for amplification can be designed [60,61], or some metabarcoding approach may be employed [62,63].

### 2.5. Analytical Methods

The proper and documented validation or verification of all protocols used for forensic species and identity testing of non-human samples should follow at least the minimum requirements [64,65,66]. These sources outline several key recommendations and standards/guidelines intended to enhance the rigor and reliability of wildlife forensic science. There is a particular emphasis on non-human DNA analysis, including core laboratory and personnel requirements, method validation, and the use of reference data and materials.

### 2.6. DNA Extraction and Quantitation

The extraction of samples of non-human origin requires validation or verification for the efficiency of DNA recovery and the removal of inhibitors for purity, so as not to consume more evidence than is necessary. The extraction technique(s) should be robust enough to process a spectrum of non-human samples (as best as is possible), including those protected by a stabilizing agent, and to remove inhibitors that impact downstream analytical processes. Additionally, enrichment procedures, such as PCR, should overcome inhibitors, such as those performed early on in human forensic identification, by the addition of, for example, bovine serum albumin (BSA) [67]. Strategies established for human forensic samples can be applied, but may not be sufficiently resilient for some samples, such as tanned skins and hides [30,68], wastewater samples [69,70], or soil samples [71,72].

DNA quantity can be determined in an extract, ranging from generic to species-specific methods. Quantitative PCR is the method of choice as it directly assays the target species, is sufficiently sensitive, and can detect the presence of inhibitors when combined into a multiplex assay containing an internal amplification control [73,74]. Laboratories focused on a specific group of related species may benefit from a multiplex quantitation assay containing species-specific targets (e.g., mtDNA), a nuclear target(s) for genus/species resolution, and an internal positive control [75]. Some inhibitors may be difficult to remove; thus, additional or particularly robust quantitation techniques (as well as extraction methods) should be validated and implemented [76,77]. However, it should be recognized that in some countries, forensic resources are insufficient to perform quantitative PCR, and alternative quantitation or robust analytical methods can be entertained, such as those by Nanodrop, Qubit, or Tapestation.

### 2.7. Primers for DNA Barcoding

DNA barcoding identifies a species by comparing a short, specific, ubiquitous (but variable) DNA sequence(s) (the “DNA barcode”) from an unknown organism or sample to a reference library of known barcode sequences [78]. Hebert et al. defined specific criteria for a universal barcoding region (High Inter-species Variation, Low Intra-species Variation, Conserved Flanking Regions, and Short Sequence Length) and proposed that the *mitochondrial cytochrome c oxidase subunit I (COI)* gene serves as the standard barcode for animals. There are hundreds of publications describing “universal primers” for barcoding, but not all primers are universally effective [79,80], or the targeted mtDNA locus is not sufficiently variable to discriminate closely related species [81]. On the other hand, some barcoding primers have been validated for forensic use [82,83,84]. Online tools like BaTAnS (The Barcoding Table of Animal Species) [85] or FISH-FIT [86] can help select appropriate species identification methods using DNA barcoding.

### 2.8. DNA-Based Barcoding Methods

Sanger sequencing has been a gold standard for species determination [87,88,89]. However, over the past two decades, massively parallel sequencing (MPS) has gained interest for use in forensic genetics and is well-suited, because it does not require pre-target processing to sequence a species of interest, and has high throughput and sensitivity of detection [90]. Various platforms are available, such as the Illumina NovaSeq [91], semiconductor-based sequencing [92], ThermoFisher Scientific S5 [93], ONT MinION nanopore sequencing [94], PacBio sequencing [95], or hybrid approaches [96]. The MinION offers the potential for field testing [97,98,99] and on-site deployment [100]. Traditional barcoding with *cytochrome c oxidase*, *cytochrome b*, and *ribosomal subunits 12S* and *16S* can be readily performed on MPS platforms. More importantly, MPS (or whole genome sequencing strategies) can be used to analyze samples containing unknown animal DNA without the need for specific targeted enrichment reagents. The combination of MPS and metagenomics provides several benefits for species barcoding of mixed samples, such as high-throughput, the capability to analyze degraded and/or low quantities of DNA, breadth and depth of coverage, and cost-effectiveness. There also are targeted approaches that have been configured for non-human DNA testing: PCR coupled with RFLP (Restriction Fragment Length Polymorphism) [101], HRMA (High-Resolution Melting Analysis) analysis [102], species-specific qPCR (quantitative PCR) [103] and PCR assays [104], RAPD approach (Random Amplified Polymorphic DNA) [105], digital droplet PCR [106,107], SNaPshot assay [108], AFLPs (Amplified Fragment Length Polymorphisms) [109], mtDNA length polymorphisms analysis of D-loop [110], 12S rRNA 16S rRNA [111], and CR (Control Region)-mtDNA [112,113]. These methods could be used as orthogonal tests for achieving greater accuracy or confidence in primary testing systems, or when the species barcoding target is for a small group of organisms, as in the food industry [114,115,116].

### 2.9. Nuclear Inserts of Mitochondrial Genome (NUMTs)

The reliability of mtDNA-based species identification can be confounded by nuclear inserts of the mitochondrial genome (NUMTs). NUMTs and the targeted mtDNA sequence may have diverged for the same taxon [117,118], but PCR primers still may bind to both NUMTs and mtDNA targets. This phenomenon can lead to overestimating the number of species detected in a sample [117]. As long as the mtDNA genome is not degraded, exonuclease V treatment before barcoding analysis [113] could be attempted to remove [linear] nuclear DNA, including NUMTs, while the circular mtDNA molecule remains intact [119]. However, if the sample is highly degraded, this approach would digest mtDNA as well. Another approach to reduce inadvertent detection of NUMTS is to use primers that are designed to preferentially amplify mtDNA and exclude nuclear DNA [120]. Additionally, selective sample enrichment could be entertained [121]. When sequence data are obtained using MPS, appropriate bioinformatic tools can identify NUMTs [122,123]. Bioinformatic and molecular tools have been shown to be capable of distinguishing between human NUMT and mtDNA [124] and similar approaches could be used for non-human species. However, there has been little effort in cataloging NUMTs in non-human species for forensic interpretation.

### 2.10. Hybrids

False positives or obtaining inaccurate results are possible when analyzing hybrids, which are the offspring of two different but genetically related species. Hybrids can occur in nature or through human-directed breeding [125]. Therefore, when barcoding results are based solely on mtDNA typing, it should be recognized that the resulting database match reflects mtDNA only (i.e., maternal origins) and possible hybrids may not be detected [126]. However, species-specific nuclear markers can be used to confirm the presence or absence of hybrids [127]. Hybridization of dogs and wolves can serve as an example of human-directed breeding with an overlap to forensic testing [128]. (The wolf (*Canis lupus*) is protected under CITES (listed in Appendix II of CITES document). This protection means that the international trade of wolves (including live animals, parts, and derivatives) is allowed, but it is controlled to ensure that the trade is not detrimental to the species’ survival in the wild. A CITES export permit is required for any international trade, and it will only be issued if the relevant authorities are satisfied that the transaction is not harmful to the wild population. It is important to note that a CITES listing is for international trade. National and regional protections, such as those under the U.S. Endangered Species Act or the EU Habitats Directive, can provide additional, and often stricter, protections. The conservation status of, for example, wolves can vary by region.) Not surprisingly, there are studies describing protocols for the differentiation of Pure Wolves, Dogs, and Wolf-Dog hybrids using SNP [129,130] or STR typing [131].

**Databases**

Taxon coverage of reference sequences is far from complete for genus or species-level identification. Given the nature of criminal and civil investigations, it is likely that a laboratory will encounter an unknown species in a forensic sample that is not in a reference database [132]. These databases, even the large-scale ones like GenBank [133] or BOLD, vary in completeness and accuracy, depending on the mtDNA locus and group of organisms studied. In addition to the “classical” barcoding databases, there are several projects, like the Darwin Tree of Life project [134,135] and Barcode UK [136], as well as regional activities (BioAlfa [137] (Costa Rica), Norwegian Barcode of Life (NorBOL) [137], Finnish Barcode of Life (FinBOL) [138], Austrian Barcode of Life [139] (ABOL), Smithsonian DNA Barcode Projects [139,140] (USA), BULCode (Bulgaria), InBIO Barcoding Initiative [141] (Portugal)) under the project International Barcode of Life [142]. Unfortunately, some past promising database projects, like ForCyt [143], are not publicly accessible. Other databases can utilize the mtDNA analysis to infer the provenance of the source animal (e.g., lion localizer [144], loxodonta localizer [145]). Missing and inaccurate records can cause false-negative results. Another aspect worth consideration, so as not to overstate the strength of evidence, is the degree of sharing of records between databases. Nakazato analyzed the relationship between the two primary public databases for DNA barcode data: the Barcode of Life Data System (BOLD) and GenBank. He determined that 11% of all COI barcode records on BOLD originated from GenBank. In contrast, 75% of the COI barcodes on GenBank were derived from BOLD [146].

Studies have demonstrated that the accuracy of DNA barcoding depends heavily on the quality of the reference databases. The error rate with sequence data is challenging, especially for older entries, to quantify and varies greatly depending on the particular group of organisms, the type of error, the methods used to sequence the sample, the database being examined, and the quality control of the submitting laboratory. The errors that exist are a combination of biological realities (cryptic species, incomplete reference libraries), technical limitations (non-standardized methods, varied platforms and chemistries, and varied bioinformatic tools, all of which are continuously evolving), and human factors (misidentification, contamination, and data entry errors) [147,148,149,150]. With the development of MPS techniques, many DNA barcode sequences have been produced and stored in online databases. Still, their degree of validity, accuracy, and reliability has not been extensively investigated [150,151]. The Organization of Scientific Area Committees (OSAC) proposed standard 2021-S-0006—“Standard for Use of Genbank for taxonomic assignment of wildlife”—for databases for species identification in wildlife [152]. The core function of the OSAC standard is to ensure the results obtained from using GenBank in forensic DNA analysis are valid, reliable, and reproducible. The standard addresses two main areas: Requirements for Sequence Comparison and Requirements for Taxonomic Assignment.

Some gaps in species coverage can be addressed by incorporating the submission of sequence data derived from other primary sources, such as the research/academic community. The published literature could be made accessible to increase the range of representation geographically, individually, and taxonomically. However, the quality of the data may be lower than what is achieved in, for example, human forensic databases. A balance may be needed between allowing access to some data that may not be “perfect” as opposed to no available data, and some additional data curation tools could be developed to enhance data quality. Additionally, incentives, such as in the form of grants, could be considered for improved collaboration among laboratories to share samples and participate in and agree upon inter-laboratory analytical tests with reference standard sequences and markers.

### 2.11. Individual Identification in Non-Human Forensic DNA Testing

Most often, non-human identity testing focuses on species-level resolution. However, there are cases in which greater resolution, i.e., individualization approaches, is needed. For example, in 2003, a dispute arose between Canada and the US regarding the provenance of a BSE-positive cow. The consequences would impact the beef industry in one of these countries. Through parentage testing using microsatellites, it was determined that the cow originated from Canada [153,154]. Another example is the comparison of tusks (elephant) or horns (rhinoceros) to recovered carcasses in criminal poaching cases [7]. DNA-based individual identification of critically endangered species and for population management can be performed with STR- or SNP-based assays that can be transferred to most forensic laboratories, although MPS is just making its way into operational forensic laboratories. Examples of such applications include rhinoceros [155,156], pangolins [157], tortoises [158,159], parrots [160,161], cranes [162], tigers [163,164], giant pandas [165,166], or elephants [167,168]). The situation is more complicated in cases involving species that have not been genetically well studied. By comparing the CITES Appendix I of CITES document and available genetic studies, some species are not “scientifically covered” (e.g., Helmeted Hornbill (*Rhinoplax vigil*) or Radiated Tortoise (*Astrochelys radiata*)), even if they are the subjects of extensive illegal trade. A possible solution can be demonstrated with Cope’s Arboreal Alligator Lizard (*Abronia aurita*), which also falls into this research gap, even though it is highly sought after by collectors and is imperiled by illegal trade and habitat destruction. While there are no publications on STR or SNP panels for *Abronia aurita*, extensive research on the broader genus *Abronia* [169] could be used to mine SNP data. To fill the “identification gap” is very difficult, as law enforcement and environmental agencies usually do not have the funding for case-driven research or are bound by legal timeframes.

Any STR- or SNP-based identification should require performing a statistical evaluation of the results based on extant population data and degrees of inbreeding [3]. Such population studies require reference samples of at least 25–30 unrelated individuals [170] and likely more subjects depending on the marker system and species. The minimum number of samples is likely species dependent, based on factors such as the species’ reproductive biology, genetic diversity, and population substructure (manmade or often influenced). Kinship analysis among animals would also benefit from knowing the mutation rates of the STR alleles [171] which would increase the number of required samples to analyze. Perhaps the rates observed for human STRs may suffice as a generic starting point for other species. The logistics of obtaining the requisite sample sizes are quite problematic, mainly due to the limited number of endangered species in zoos and breeding facilities, but also due to existing shipment regulations [172]. Regardless, limitations in any statistical analysis should be appreciated and explicitly stated.

A compelling example of conservation interventions through genetic diversity comes from research on Kenyan lion populations [173]. Scientists analyzed lions’ DNA to assess the impact of their genetic health on management practices, such as relocation and fencing. Relocating lions to mitigate conflict led to the mixing of genetically distinct populations, which, while increasing localized diversity, risked loss of unique evolutionary lineages across the region. Conversely, fencing national parks diminished genetic diversity by preventing natural dispersal and gene flow, likely increasing inbreeding. This genetic evidence allowed researchers to conclude that both interventions were, in different ways, either diminishing or complicating preservation of long-term diversity, leading to recommendations for new strategies, such as controlled gene flow and limiting long-distance translocations to protect the species’ overall genetic integrity.

Another aspect that should be considered is possible extensive inbreeding leading to the loss of genetic diversity [125,174]. Inbreeding within a closed, small population tends to accelerate the loss of genetic diversity and decrease the heterozygosity of genes and forensically relevant genetic markers, potentially leading to complete homozygosity, fixation of rare alleles, and possible misidentification in inbred populations [175]. Inbreeding is not connected only with domestic animals [176,177], but also is common within the populations of highly endangered, and thus CITES-protected, species going through population size reduction and a genetic bottleneck [176,178,179].

The success of human DNA identification efforts relates to national databases, such as CODIS [180,181,182]. To date, only a few animal species databases have been developed, mostly because of their special appeal or favored domestic status. Tigris ID [183], RhODIS [155], African elephant [184,185], dogs [186,187,188], cats [189], and horses [190,191] have their DNA profiles systematically stored in databases that can be utilized for genetic identity testing purposes [191] but may not necessarily have discipline, community-wide support as do the databases employed for human identification. These databases are maintained primarily by laboratories specializing in particular animal species. The commercial availability of any animal-DNA-typing multiplexes directly results from a legal, high-volume market. Unlike the illicit and fragmented trade of wild species, the domestic animal sector can provide for a stable and recurring demand for genetic services. This market is served by commercial companies that develop and sell products to meet the needs of breeders, ranchers, pet owners, and food manufacturers. Commercially available DNA-typing multiplex kits could facilitate populating databases and the generation of centralized storage repositories, as has been seen in human DNA with EMPOP [192] and YHRD [193]. Unfortunately, funding, national/international commitment, and legal penalties are insufficient to drive standardization. For example, in Zimbabwe, stealing a goat (i.e., livestock) can result in as much as 6 years’ imprisonment. In contrast, poaching a sable antelope (worth almost a few hundred to a thousand times more than a goat) can result in a minimal fine, community service, or release on probation [28]. The lack of genetic tools for wildlife species directly results from the legal and commercial status of the species in question. The prohibition of trade for CITES Appendix I species makes a market for a commercial identification kit unfeasible, shifting the focus entirely to a research-driven or personal-passion model of forensic panel development and data sharing.

### 2.12. ISO Standards

It is necessary to note, that even if no ISO standards exist for wildlife forensic sciences, there is a number of recommendations produced by bodies involved in the development of wildlife science guidelines or standards: Society for Wildlife Forensic Sciences (SWFS); ENFSI-APST (Animal, Plant, and Soil traces working group of European Network of Forensic Science Institutes); The Technical Working Group of the Society for Wildlife Forensic Sciences SWFS TWG), which replaced the Scientific Working Group for Wildlife Forensic Sciences (SWGWILD); African Wildlife Forensics Network (AWFN); and the TRACE Wildlife Forensics Network. Accredited forensic laboratories usually work under ISO 17025 [194], but a new norm, ISO 21043 [195], is tailored for Forensic Sciences [196,197]. To date, there are no accrediting bodies operating with ISO 21043 assessments. Wildlife identification laboratories seeking certification for their work could use the program offered by the Society for Wildlife Forensic Sciences (SWFS). There are also other bodies, like the U.S. Fish & Wildlife National Forensics Laboratory, the California Department of Fish and Wildlife, UC Davis, NOAA Fisheries, the PAW Forensic Working Group, the African Wildlife Forensic Network, and the Netherlands Forensic Institute, that are directly or indirectly involved in developing standards and guidelines.

### 2.13. Legal and Ethical/Conduct Hurdles

It is essential that the sampling does not inflict unnecessary trauma on living animals. The protection of animals used for scientific purposes, as stated by Directive 2010/63/EU of the European Parliament and of the Council of 22 September 2010, must be respected. It is worth noting that ethics and legal considerations vary from country to country and even within organizations, which can slow down information and sample exchange as well as the access and harmonization of analytical techniques and reference standards. Another regulated area that requires compliance is the shipping of CITES-protected organisms’ samples (which may include PCR products), e.g., for collaborative studies. This restriction complicates research development, the validation and implementation of methods, proficiency testing, and collaborative exercises. Thus, domesticated or common animals are used as surrogates [198]. Using mimicked samples or extracted, amplified, and/or synthetic DNA [199] could overcome, to some degree, this bureaucratic but necessary burden [200]. Alternatively, democratizing capabilities to enable work in less-resourced countries could be sought, so collaborative work can be sought.

## 3. Discussion

The challenges facing non-human animal identity testing are discussed briefly herein, particularly when addressing the vast taxonomic diversity and the wide array of biological samples that may be and have been encountered in non-human identity genetics investigations. These samples, which can range from animal parts in traditional medicine to taxidermy specimens and eDNA, can contain degraded or low-quantity DNA, which may be contaminated with chemical inhibitors or human DNA from field agents, investigators, and, at times, first responders, and may be comingled with other species materials. There are limitations to attempting to use methods and tools designed specifically for human forensics, which may be inadequate for the diverse and complex nature of such evidence. However, MPS technologies may be a solution as they can democratize analyses for most animal species (as well as for humans). There are also issues with data interpretation, such as NUMTs, population substructure, and minimal population data, and these limitations should be stated explicitly when reporting results. Nonetheless, the extensive experience gained from human identity testing can be effectively adapted to some degree to other animal species. Although not addressed herein, greater methodological innovation is required for microbial and plant species that do not conform to a similar sexual reproduction strategy on which human forensic DNA interpretation is based.

De Bruyn and co-authors [7] recently addressed wildlife forensic DNA challenges encountered in South Africa. They described region-specific administrative and infrastructure gaps and challenges in South Africa (underfunding, court prioritization, and tracking impact) alongside technical barriers relevant to its unique biodiversity (endemism, localized hybridization issues). Our review presented challenges with a more global perspective; however, many of the challenges are similar. The two perspectives should be appreciated, and differences should not be construed as conflict, but instead they complement each other and show the varied and sometimes specific needs that must be met across the various countries and laboratories carrying out nonhuman species identification work (Table 2).

A central theme is the gap in available resources necessary for genetic identification, for example, of endangered species. While genetic tools exist for some high-profile species, many CITES-listed organisms lack specific, validated genetic markers and comprehensive reference databases. Therefore, some high-level strategic and technical improvements across the field are recommended:

- **Improve Sampling Procedures:** For composite artifacts like taxidermy, sub-sample an item (bearing in mind constraints on damaging certain artifacts) to reduce the risk of false-positive or false-negative results. Use sampling kits containing reagents that stabilize nucleic acids and inactivate infectious agents (when appropriate), which are crucial for field-collected samples without cold-chain storage. Take special care to avoid contamination with human DNA, particularly when collection and/or sampling is performed by non-specialized personnel.
- **Advance Laboratory Protocols:** Develop and validate DNA extraction and inhibitor removal protocols specifically for non-human samples (such as those undertaken in microbial forensics applications), as human forensic methods may be insufficient for the task. Employ, when feasible, qPCR with an internal amplification control to accurately measure the target DNA and determine the impact of inhibitors in a sample. Produce robust enrichment kits that may be able to work in the presence of inhibitors at least to some degree. Acknowledge the limitations of mtDNA-based typing. Use, when possible, species-specific nuclear markers to confirm or exclude the presence of hybrids and sample mixtures. Embrace high-throughput sequencing methods to improve detection and sensitivity over current or traditional approaches. Acknowledge and explicitly state limitations in any statistical calculations and inferences.
- **Enhance Standards and Databases:** Develop and validate sampling and diagnostic kits specifically for non-human genetic identification, as current ISO standards, like ISO 18385, do not cover non-human DNA contaminants. Adhere to standards and recommendations from bodies like SWFS and ENFSI-APST, which provide guidelines for the field. If the restriction of shipping CITES-protected samples cannot be overcome, then pursue collaborative studies and/or use mimicked DNA. Alternatively, develop memoranda of understanding with in-country zoos or museums to obtain a number, albeit limited, of samples. Recognize that online genetic databases like GenBank and BOLD have varying levels of completeness, overlap with entries, and can contain errors, which should be considered during the development and validation of assays and during analysis. Genetic testing and database searches also serve as deterrents for potential offenders who may know they can be easily linked to crimes.
- **Adopt a Multidisciplinary Approach:** The spectrum of relevant samples in wildlife crime cases is vast. Additional methods, such as profiling of volatilomes [201], mass spectrometry [202], hair and feather morphology [203,204], osteology [205,206], radiocarbon dating [207], and the analysis of stable isotopes [208,209,210], to name a few, may provide faster answers than DNA analysis, or at least provide additional support for findings.

## 4. Conclusions

Modern non-human (animal) genetic identity testing is an interdisciplinary field dedicated to supporting law enforcement, civil legal issues, and conservation efforts. There are many challenges to achieving effective and sustainable animal species identification. Advances in genetic testing can substantially help support investigations and prevent future crimes in several ways. Overall, innovation and collaboration in animal forensic genetics will be essential in combating wildlife crime. A rapid DNA or field-forward profiling system can quickly link cases, uncover a network of illegal traders, and thus prevent them from committing further crimes. Additionally, enhanced databases of animal DNA profiles support species and individualization efforts and provide resources for better studies on population substructure and management strategies. These general efforts strengthen capacity-building and leverage resources to better serve society to uphold wildlife protection laws, bolster conservation initiatives, support investigations, and protect the public.

The recommendations outlined above for appreciating limitations of and improving non-human genetics identity testing also carry critical implications for conservation genetics, primarily by identifying the substantial resource gap that currently exists for investigating and protecting CITES-listed and endangered species. The push to advance laboratory protocols directly supports conservation efforts by promoting development and standardization, as best as is applicable, species-specific nuclear markers, which are vital to confirm or exclude hybrids and mixtures. There are inherent limitations of relying solely on mtDNA typing. Moreover, validating robust DNA extraction, inhibitor removal protocols, and inhibitor-resistant enrichment and typing methods better permits degraded or chemically treated samples, such as processed animal parts in traditional medicine or taxidermy specimens, to yield viable genetic material, making older or poorer quality evidence useful for genetic testing. To overcome the difficulty of acquiring adequate reference samples for calculating population statistics and genetic diversity estimates, enhanced standards and databases are recommended by pursuing collaborative studies, developing memoranda of understanding with in-country zoos and museums, and incentivizing the exchange of protocols and resources. A focus on data quality and sample access is critical, as accurately identifying individual animals and understanding population substructure are confounded by issues, such as extensive inbreeding and loss of genetic diversity common in small, endangered populations. Overall, these desired strategic improvements aim to provide accurate, validated genetic tools necessary for effective species protection, supporting conservation initiatives, and aiding law enforcement in combating wildlife crime.

## Figures and Tables

**Table 1:** Brief comparison of human and animal fields of forensic DNA testing.

Discipline	Primary Objectives	Examples of Typical Samples
Human DNA Forensics	Individual identification of a person of interest, victim, or missing person; parentage and kinship analysis; genealogical leads in criminal cases.	Blood, saliva, semen, hair with follicle, tissue, hair shaft, skin cells (touch DNA), bone, teeth.
Animal DNA Forensics	Species identification; determination of geographic origin; individual identification (less common); parentage and kinship analysis; confirmation of captive-bred vs. wild status.	Blood, tissue, hair, bone, feces, processed animal products (meat, leather, horn, ivory), vomit, stomach contents.

**Table 2:** Comparison of recommendations for wildlife DNA forensics from a South African and a general application perspective.

Focus Area	A Septennium Review of Wildlife Forensic DNA Analysis in South Africa	General Application Perspective
Tracking Judicial Impact	Unique recommendation: Track successes and challenges when forensic case reports are submitted to courts to understand the impact of genetic evidence on convictions and sentences, which is essential for obtaining further funding.	This aspect is not addressed, although it should be supported. Focus instead is on the scientific and technical steps required for maximizing typing success, and thus, before court submission.
Addressing Ambiguous Taxonomy	Highlights challenges with changing or unclear taxonomy for specific South African species (e.g., African elephants, giraffes) and the need for validated technologies that reflect these changes for forensic distinction.	Discusses the issue of hybridization and NUMTs. Does not focus on specific taxonomic reclassifications or legislative lags for endemic species as a primary challenge. A good recommendation for those working on specific species and distinguishing between close species.
Contamination/Lab Protocols	Focuses generally on training to avoid contamination.	Recommendations for laboratory protocols: Develop and validate DNA extraction and inhibitor removal protocols specifically for non-human samples, employ qPCR with an internal amplification control to accurately measure target DNA and detect inhibitors, discuss the use of heat-labile dsDNase to mitigate non-human contamination in reagents, and consider contaminating-human DNA as a potential investigative lead.
Standardization and Resources	Mentions following ISFG standards and addressing chronic underfunding.	Recommendations for standardization: Develop kits specifically for non-human identification, noting that ISO 18385 does not cover non-human DNA contaminants; suggest alternatives for obtaining CITES samples (collaborative studies, mimicked DNA, Memoranda of Understanding with zoos) if shipping restrictions cannot be overcome. Also notes under funding issues.
Multidisciplinary Approach	Not a primary recommendation, but necessity is implied by the technical hurdles.	Recommendation to adopt a multidisciplinary approach, utilizing methods like profiling of volatilomes, mass spectrometry, hair morphology, osteology, and radiocarbon dating as additional or orthogonal tests.

## Data Availability

No new data were created or analyzed in this study.
